# Comparison of NSGA-II, MOALO and MODA for Multi-Objective Optimization of Micro-Machining Processes

**DOI:** 10.3390/ma14175109

**Published:** 2021-09-06

**Authors:** Milan Joshi, Ranjan Kumar Ghadai, S. Madhu, Kanak Kalita, Xiao-Zhi Gao

**Affiliations:** 1 Department of Applied Science and Humanities, MPSTME SVKM’S Narsee Monjee Institute of Management Studies, Shirpur 425 405, India; milan.joshi@nmims.edu; 2 Department of Mechanical Engineering, Sikkim Manipal Institute of Technology, Sikkim Manipal University, Majhitar 737 136, India; 3 Department of Automobile Engineering, Saveetha School of Engineering, Saveetha Institute of Medical and Technical Sciences, Chennai 602 105, India; madhu.sse@saveetha.com; 4 Department of Mechanical Engineering, Vel Tech Rangarajan Dr. Sagunthala R&D Institute of Science and Technology, Avadi 600 062, India; 5 School of Computing, University of Eastern Finland, FI-70210 Kuopio, Finland; xiao-zhi.gao@uef.fi

**Keywords:** process optimization, metaheuristics, ant lion optimization, dragonfly algorithm, NSGA

## Abstract

The popularity of micro-machining is rapidly increasing due to the growing demands for miniature products. Among different micro-machining approaches, micro-turning and micro-milling are widely used in the manufacturing industry. The various cutting parameters of micro-turning and micro-milling has a significant effect on the machining performance. Thus, it is essential that the cutting parameters are optimized to obtain the most from the machining process. However, it is often seen that many machining objectives have conflicting parameter settings. For example, generally, a high material removal rate (MRR) is accompanied by high surface roughness (SR). In this paper, metaheuristic multi-objective optimization algorithms are utilized to generate Pareto optimal solutions for micro-turning and micro-milling applications. A comparative study is carried out to assess the performance of non-dominated sorting genetic algorithm II (NSGA-II), multi-objective ant lion optimization (MOALO) and multi-objective dragonfly optimization (MODA) in micro-machining applications. The complex proportional assessment (COPRAS) method is used to compare the NSGA-II, MOALO and MODA generated Pareto solutions.

## 1. Introduction

In this new age of industrialization, the conventional and non-conventional manufacturing processes are undergoing revolutionary changes in their capability to fabricate micro-components with extreme precision [1]. There is a growing need to manufacture micro-scale pumps, small valves, and various micro components of electronics and medical applications [2]. The micro-components demand high accuracy and a high production rate. For the fabrication of these tiny elements, a potential machining process needs to be selected. Micro-turning is one of the suitable machining processes widely used to manufacture these types of components [3]. The micro-manufacturing processes are the extension of the traditional manufacturing process [4]. The working principle of micro-turning operation is the same as the traditional turning operation. The tools used for machining are in the range of 50–1000 µm. The micro-turning operation can fabricate 3-D structures on a micro-scale. This machining process is influenced by several factors, namely speed, feed rate, depth of cut, dimension, tool cutting force, work materials, etc. Hence, the fabrication of micro components with required dimensional accuracy is a great challenge [5]. Micro-milling is also an important micro-machining process and can produce complex 3-D structures [6]. Proper selection of process parameters is one of the ways to overcome this challenge.

The surface roughness is a very important factor as it directly influences the tribological performance of the machined component. Thus, the smoother machined surface needs to produce by proper cutting conditions to enhance quality and performance [7]. Along with that, a higher material removal rate (MRR) is required to increase the production rate. These two objectives are conflicting in nature. To achieve better performance, the optimal parameter setting is essential. This can be achieved by the utilization of optimization techniques [8]. Researchers have applied various optimization techniques for the selection of proper machining conditions [9,10,11,12,13]. Kibria et al. [14,15] developed mathematical models using response surface methodology for micro-turning operation. Analysis of variance (ANOVA) analysis was performed to check adequacy. Pradhan and Bhattacharyya [16] used response surface methodology for modelling and optimization of micro- electro-discharge machining (µ-EDM) process. Dhupal et al. [17] developed mathematical relations and optimized process parameters of the micro-grooving process using response surface methodology (RSM). Mia et al. [18] developed mathematical relations and investigated the influence of machining parameters using RSM. Chen et al. [19] developed a mathematical model using RSM and utilized this model as a fitness function in the genetic algorithm-particle swarm optimization (GA-PSO) hybrid technique to optimize the plastic injection moulding process. Bharti et al. [20] utilized the non-dominated sorting genetic algorithm II (NSGA-II) technique to optimize the machining process. Pasandideh et al. [21] carried out a comparative study between NSGA-II and multi-objective particle swarm optimization (MOPSO) for optimizing bi-objective multi-product EPQ model. Slightly better performance was found for MOPSO. Majumder et al. [22,23] developed mathematical models and adequacy checks by ANOVA test. The developed models were used in conjunction with MOPSO to optimize the EDM process. Similarly, Prakash et al. [24] also used RSM models with MOPSO to find optimal parameter settings. Mirjalili introduced Ant Lion Optimization (ALO) [25] and a Dragonfly algorithm (DA) [26] to solve real-life engineering problems. Dubey et al. [27] demonstrated the applicability of the ALO algorithm in real-world problems, and it is found that this is proficient in producing encouraging solutions. Wang et al. [28] utilized multi-objective dragonfly optimization (MODA) to optimize electrical power systems, and it is observed that this is an effective tool for optimization. Optimization algorithms find widespread applicability in diverse fields [29,30,31].

From the literature, it is observed that RSM is a widely used and well-established technique to formulate mathematical relations between input process parameters and the output responses. These mathematical or empirical relations developed using the experimental data can be used as objective functions in the optimization process. As evident from the brief section of literature discussed above, single-objective optimization is mostly carried out by researchers. However, single-objective optimization is not necessarily helpful in real-life applications as multiple responses must be looked at simultaneously to assess the effectiveness or suitability of a machining process. Most research conducted on multi-objective optimization has generally considered some variant of NSGA or MOPSO. However, NSGA was proposed by Srinivas and Deb in 1995 [32] and MOPSO by Coello and Lechuga in 2002 [33]. Thus, there is tremendous need for application of more recent and powerful multi-objective optimization algorithms to machining problems. This could lead to better optimized solutions which would translate into cost saving and efficiency increment in the real-world situations. It is also observed from the literature search that there are very limited studies carried out on the comparison of various metaheuristics for multi-objective optimization of machining processes. Further, no suitable studies on the implementation of MOALO and MODA for multi-objective optimization of machining processes are found. Thus, in the present study, NSGA-II, MOALO and MODA are used for multi-response Pareto optimization of micro-machining processes. Two examples from the literature on micro-turning and micro-milling are selected to demonstrate and compare the three metaheuristics.

## 2. Multi-Objective Optimization

In this section, three different metaheuristic multi-objective algorithms are described. These algorithms simultaneously optimize multiple objectives to generate a set of non-dominated solutions known as the Pareto front.

### 2.1. Non-Dominated Sorting Genetic Algorithm (NSGA-II)

The NSGA-II is an advanced multi-objective optimization algorithm that efficiently handles multi-objective optimization problems. This algorithm was proposed by Deb et al. [34]. This algorithm is implemented based on the idea of the selection of the dominant among all solutions. This algorithm is described in brief as follows [35]:

(I) Initialization of population $P_{o}$ of size N using a uniform distribution.
(II) Generate new offspring population $Q_{t}$ by utilizing binary tournament selection which is based on crowding comparison operator, crossover, and mutation operation on the parent population ($P_{t}$). Here, t denoted the number of generations. The entire population ($R_{t}$) is the combination of offspring population ($Q_{t}$) and its parent population ($P_{t}$).
(III) Non-dominated fronts of different objective functions are obtained by performing a fast non-dominated sorting approach on the entire population ($R_{t}$).
(IV) Generate a new parent population ($P_{t+1}$) from the obtained fronts.
(V) This process is continued until the maximum number of iterations is reached.

### 2.2. Multi-Objective ant Lion Optimization (MOALO)

The algorithm of Ant Lion Optimization (ALO) was inspired by the unique hunting behaviour of antlions [25,36,37]. It solves the optimization problem by considering the random walk of ants, constructing traps, entrapment of ants in traps, catching ants, and re-building of traps. It mimics the relationship between antlions and the trapping of ants. To model this relationship, ants are needed to be move over the search space, and antlions are allowed to hunt them using traps. The ants move stochastically in search of food. The random movement of ants are mathematically denoted as Equation (1),

(1) $$X\left(t\right)=\left[0,\;cs\left(2r\left(t_{1}\right)-1\right),\;cs\left(2r\left(t_{2}\right)-1\right),\ldots ,\;cs\left(2r\left(t_{n}\right)-1\right)\right]$$

where cs is the calculative sum, n is the maximum number of iterations, t is the step of random walk, and $r\left(t\right)$ is the stochastic function defined as Equation (2),

(2) $$r\left(t\right)=\left\{\begin{array}{c} 1,\;if\;rand>0.5\\ 0,\;if\;rand\leq 0.5 \end{array}\right.$$

where rand is a uniform distribution random number, generated within the interval [0, 1]. The random walk of ants is maintained within the search space and normalized using Equation (3),

(3) $$X_{i}^{t}=\frac{\left(X_{i}^{t}-a_{i}\right)\times \left(d_{i}^{t}-c_{i}^{t}\right)}{\left(b_{i}-a_{i}\right)}+c_{i}^{t}$$

where $a_{i}$ and $b_{i}$ are the minimum and maximum random walk, respectively of the *i*th variable. $c_{i}^{t}$ and $d_{i}^{t}$ are the minimum and maximum value of the *i*th variable at the *t*th iteration.

The random walk of the ants is affected by the pits of antlions. The entrapment of ants is found by changing the random walks around the antlions, and this is mathematically expressed as Equations (4) and (5),

(4) $$c_{i}^{t}=Antlion_{j}^{t}+c^{t}$$

(5) $$d_{i}^{t}=Antlion_{j}^{t}+d^{t}$$

where $c^{t}$ is the minimum of all variables at *t*th iteration, $d^{t}$ indicates the vector including the maximum of all variables at *t*th iteration, $c_{i}^{t}$ is the minimum of all variables for *i*th ant, $d_{i}^{t}$ is the maximum of all variables for *i*th ant. $Antlion_{j}^{t}$ shows the position of the selected *j*th antlion at *t*th iteration. Large size antlions build large size pits to increase the possibilities to slide the ants into the pits. For sliding into the pits, the boundaries of random walks need to be reduced, and it expresses mathematically as Equations (6) and (7),

(6) $$c^{t}=\frac{c^{t}}{I}$$

(7) $$d^{t}=\frac{d^{t}}{I}$$

where $I=1+10^{w}\frac{t}{maximum\;iteration}$, t is the current iteration, and w is a constant and the values are given as follows,

(8) $$w=\{\begin{array}{c} 2\;when\;t\;>\;0.1\;*\;maximum\;iteration\\ 3\;when\;t\;>\;0.5\;*\;maximum\;iteration\\ 4\;when\;t\;>\;0.75\;*\;maximum\;iteration\\ 5\;when\;t\;>\;0.9\;*\;maximum\;iteration\\ 6\;when\;t\;>\;0.95\;*\;maximum\;iteration \end{array}$$

Finally, an ant is trapped into the bottom of the pit and caught by the antlion. After this stage, the antlion changes its position and re-construct pits for catching a new ant. The mathematical equation in this regard is,

(9) $$Antlion_{j}^{t}=Ant_{i}^{t}\;if\;f\;\left(Ant_{i}^{t}\right)>f\left(Antlion_{j}^{t}\right)$$

where t is the current iteration, $Ant_{i}^{t}$ is the position of *i*th ant at *t*th iteration and $Antlion_{j}^{t}$ is the position of *j*th antlion at *t*th iteration. In evolutionary algorithms, elitism is an important characteristic. In this process, the best antlion is considered as an elite and stored. It is conducted by using Equation (10),

(10) $$Ant_{i}^{t}=\frac{R_{A}^{t}+R_{E}^{t}}{2}$$

where $R_{A}^{t}$ and $R_{E}^{t}$ are the random walk around the antlion and elite at *t*th iteration, respectively. During each iteration, if it is found that the current antlion is fitter than the existing elite, then an update of the elite is conducted. An archive is used in MOALO to store the Pareto optimal solutions. To improve the Pareto optimal solution distribution and diversity in the archive, niching is adopted.

### 2.3. Multi-Objective Dragonfly Algorithm (MODA)

A dragonfly is a beautiful, small insect, having unique swarming behaviours for hunting and migration [26,38,39]. These two behaviours of the Dragonfly are also termed as static and dynamic swarm behaviour. Dragonflies form small sub-group and fly over locality by changing the steps in a static swarm. In migratory (dynamic) swarms, dragonflies create large groups and move over long distances. The dragonfly algorithm (DA) is based on five different behaviours of the dragonfly in a swarm, namely separation, alignment, cohesion, attraction towards the food and distraction from enemy sources. The separation behaviour is mathematically expressed as Equation (11),

(11) $$S_{i}=-\overset{N}{\underset{j=1}{\sum }}X-X_{j}$$

where X is the position of current individual, $X_{j}$ is the position of the *j*th neighbouring individual, and N is the total number of neighbouring individuals.

Alignment behaviour is mathematically denoted as Equation (12),

(12) $$A_{i}=\frac{\sum_{j=1}^{N}V_{j}}{N}$$

where $V_{j}$ is the velocity of the *j*th neighbour.

The tendency of flying toward the neighbouring centre of mass refers to the cohesion behaviour. It is mathematically expressed as Equation (13),

(13) $$C_{i}=\frac{\sum_{j=1}^{N}X_{j}}{N}-X$$

Attraction and distraction refer to the tendency of dragonflies to fly towards the food and to fly away from an enemy, respectively. These are mathematically modelled as Equations (14) and (15),

(14) $$F_{i}=X^{+}-X$$

(15) $$E_{i}=X^{-}+X$$

where $X^{+}$ and $X^{-}$ represent the position of food and enemy.

The above five principles influence the behaviour of dragonflies. The position of the dragonflies in the search space are updated using two vectors: step vector $\left(\Delta X\right)$ and position $\left(X\right)$. The movement of dragonflies is represented by a step vector. The mathematical model is represented as Equation (16),

(16) $$\Delta X_{t+1}=\left(w_{1}S_{i}+w_{2}A_{i}+w_{3}C_{i}+w_{4}F_{i}+w_{5}E_{i}\right)+v\Delta x_{i}$$

where Δx is the step size, v is the inertia weight, and the weight value of $w_{1},\ldots ,w_{5}$ are assigned for each of the operators. The values of weight are enabled to obtain different exploration and intensification behaviours.

The position of an individual in search space is updated as per Equation (17),

(17) $$x_{t+1}=x_{t}+\Delta x_{t+1}$$

Levy Flight equation [40] is used to update the position if no dragonfly exists in neighbourhood radius, and the equation is expressed as Equation (18),

(18) $$x_{t+1}=x_{t}+levy\left(d\right)\times x_{t}$$

where *d* is the dimension of the position vector.

An archive is used in MODA to store the Pareto optimal solutions.

## 3. Multi-Criteria Decision Making with COPRAS

Multi-criteria decision making (MCDM) methods are often used to determine compromise solutions in situations where multiple criteria need to be taken into account. Complex Proportional Assessment (COPRAS) is an MCDM method developed by Zavadskas and Kaklauskas [41] in 2011.

Any MCDM problem that contains m alternatives and n criteria can be expressed in form of a decision matrix D.

(19) $$D=\left[\begin{array}{cccc} x_{11} & x_{12} & \cdots & x_{1n}\\ x_{21} & x_{22} & \cdots & x_{2n}\\ \vdots & \vdots & \ddots & \vdots \\ x_{m1} & x_{m2} & \cdots & x_{mn} \end{array}\right]$$

A relative degree of importance must be assigned to each criterion. In MCDM terminology, this is called weight allocation. The weight vector can be expressed as per Equation (20),

(20) $$w_{j}=\left[\begin{array}{ccc} w_{1} & \ldots & w_{n} \end{array}\right],\;\mathrm{such}\;\mathrm{that}\;\sum_{j=1}^{n}\left(\begin{array}{ccc} w_{1} & \ldots & w_{n} \end{array}\right)=1$$

All criteria can be classified into cost (C) and benefit (B) criteria. Cost criteria are those that require minimization or lower values, which are desired. Similarly, benefit criteria are those that need to be maximized, i.e., higher values are desired. Since different criterion values are in different scales, all of them must be converted to a common scale. This process is referred to as normalization. This is obtained as per Equation (21),

(21) $$n_{ij}=\frac{x_{ij}}{\sum_{j=1}^{n}x_{ij}}$$

Next, the weighted normalized matrix is calculated as per Equation (22),

(22) $$N_{ij}=w_{j}*n_{ij}\;\mathrm{where}\;i\in \;\left[1,m\right]\;\mathrm{and}\;j\in \;\left[1,n\right]$$

The sum $B_{i}$ of the benefit criteria values is then calculated as per Equation (23),

(23) $$B_{i}=\overset{k}{\underset{j=1}{\sum }}N_{ij}$$

The sum $C_{i}$ of the cost criteria values is then calculated as per Equation (24),

(24) $$C_{i}=\overset{m}{\underset{j=k+1}{\sum }}N_{ij}$$

where k are the benefit criteria and $\left(m-k\right)$ are the cost criteria.

The relative significance $Q_{i}$ of each alternative is then calculated as per Equation (25),

(25) $$Q_{i}=B_{i}+\frac{min\left(C_{i}\right)\cdot \sum_{i=1}^{n}C_{i}}{C_{i}\cdot \sum_{i=1}^{n}\left(\frac{min\left(C_{i}\right)}{C_{i}}\right)}$$

Finally, the utility degree for each alternative is determined as per Equation (26),

(26) $$UD_{i}=\frac{Q_{i}}{max\left(Q_{i}\right)}\times 100\%$$

## 4. Results and Discussion

### 4.1. Example 1: Optimization of Micro-Turning Process Parameters

In this case study, a micro-turning example from Kumar [42] is used. Kumar [42] carried out micro-turning of C360 Copper alloy using a Tungsten carbide insert. He used a Taguchi L27 orthogonal array for the design of experiments and a polynomial regression approach to generate empirical mathematical relations between the responses and the process parameters. MRR and surface roughness ($R_{a}$) are considered as the responses while cutting speed (N), feed rate (f), and depth of cut (D) are considered as the process parameters.

#### 4.1.1. Mathematical Modelling

In this paper, the mathematical relations from [42] are made more robust by analysing them using ANOVA and then using the stepwise elimination method to remove the statistically insignificant terms. The modified mathematical relations are presented in Equations (27) and (28).

(27) MRR=0.0437−0.000024N−0.003923f−0.000856D+0.000002Nf+0.00000049ND+0.000076fD

(28) $$R_{a}=-0.002238+0.000017N-0.000047f-0.000040D+0.000009fD-0.000000004N^{2}+0.000001D^{2}$$

ANOVA for the mathematical relations in [42] is computed and presented in Table 1. From Table 1, it is observed that in the MRR model, $N^{2}$, $f^{2}$ and $D^{2}$ terms have a *p*-value greater than the threshold value of 0.1. Thus, these three terms are statistically insignificant to the model and are responsible for artificially inflating the $R^{2}$ of the model. Similarly, in the $R_{a}$ model, Nf, ND and $f^{2}$ are statistically insignificant.

**Figure 1:**
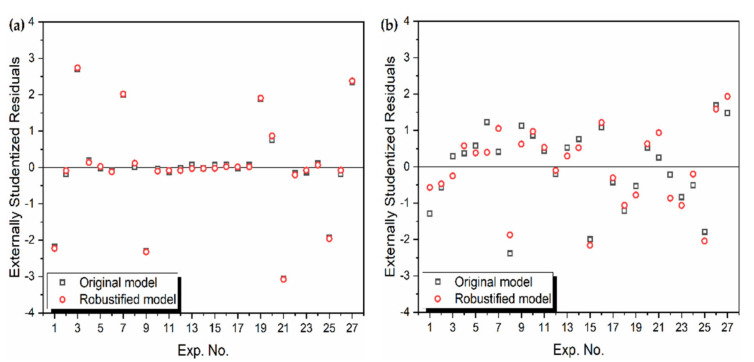
Externally studentized residuals of the previous [42] and current models for (**a**) MRR (**b**) $R_{a}$.

Thus, the stepwise elimination method is used and the ANOVA of the robust mathematical models is presented in Table 2. It is seen that all the model terms in Table 2 have *p*-values much smaller than 0.1, indicating the adequacy of the developed models. The comparison of the various accuracy metrics for the previous [42] and current model is presented in Table 3. Further, Figure 1 shows the comparison of the residuals of the models. For MRR, as seen in Figure 1a, the improvement is minor. However, as seen from Figure 1b, for the $R_{a}$ model there is a significant improvement. Thus, these developed models are used as objective functions for multi-objective optimization in the next section.

#### 4.1.2. Multi-Objective Optimization

It is well known that MRR and $R_{a}$ are two important responses of turning operation, which are conflicting in nature. Higher MRR and lower $R_{a}$ is always desired for turning operation. Hence, optimal parameter settings need to be found for obtaining high MRR and low $R_{a}$. The two objective functions can be stated as,

Objective 1 = Maximize MRR

Objective 2 = Minimize *R_a_*

The optimization of micro-turning process parameters is performed by implementing three different intelligent optimization techniques. To perform the multi-objective optimization of process parameters, i.e., cutting speed, feed rate, and depth of cut with respect to MRR and *R_a_*, the following boundary conditions are used.

(29) $$\begin{array}{c} 1000\;\leq \;N\;\leq \;2500\;\mathrm{rev}/\min\\ 2\;\leq \;f\;\leq \;20\;µm/\mathrm{rev}\\ 10\;\leq \;D\;\leq \;100\;µm \end{array}$$

For the sake of ease in comparison of the performance of the three metaheuristic algorithms, the total function evaluation is kept the same. In all the three algorithms, i.e., NSGA-II, MOALO and MODA, the number of search agents or population size is kept as 100. This is then iterated for 500 generations. Thus, the total function evaluations are 50,000. The maximum archive size of the non-dominated solutions is considered to be 500.

**Figure 2:**
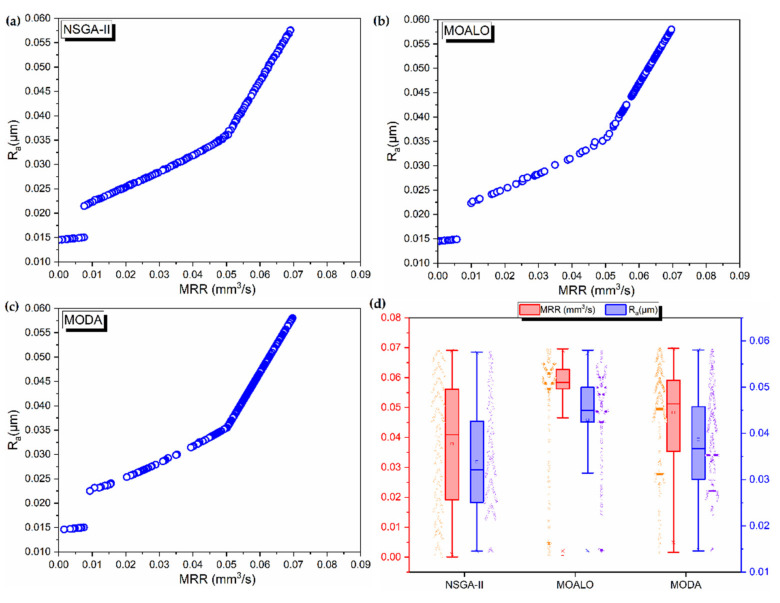
Pareto fronts for optimization of micro-turning process using (**a**) NSGA-II (**b**) MOALO (**c**) MODA. (**d**) Box plot showing the spread of the Pareto fronts.

The Pareto fronts generated by the three algorithms are presented in Figure 2. It is seen that a small discontinuous zone is present in all the Pareto fronts at low MRR and low $R_{a}$ combination, indicating that no non-dominated solutions are present in that region. Further, the NSGA-II is seen to be better in the continuity of the Pareto fronts as compared to MOALO and MODA. Especially in the case of MODA, two more distinct breaks in the Pareto front are seen. The spread of the Pareto fronts is analysed by using box plots in Figure 2d. The spread is closer to normal distribution for NSGA as compared to MODA and MOALO.

#### 4.1.3. Comparison of the Metaheuristics

The numerical experiments are carried out on a Dell Inspiron 15-3567 series windows system with Intel(R) CoreTM i7-7500U CPU @2.70 GHz, Clock Speed 2.9 Ghz, L2 Cache Size 512 and 8 GB ram. To account for the stochasticity of these algorithms, Pareto optimization by each algorithm is carried out for 10 independent trials. The average computational time for each trial is found to be approximately 1908 (±125) s, 972 (±38) s and 1366 (±63) s, respectively, for NSGA-II, MOALO and MODA. The values within the bracket indicate the standard deviation of 10 trials.

Since no Pareto optimal solutions from the literature are found in this case study, the NSGA-II solutions are considered as the benchmark solution. The MOALO and MODA solutions are contrasted against the NSGA-II solutions by using various convergence and diversity measuring metrics such as generational distance (GD), inverted generational distance (IGD), convergence metric (CM) and spread (SP) in Table 4. Both MOALO and MODA solutions are observed to be superior to the NSGA-II solutions.

To further analyse the performance of the three metaheuristics, COPRAS is used. Three test case scenarios are considered where three different weights are allocated to the MRR objective. One optimal solution as per COPRAS is predicted from the Pareto front of each metaheuristic and is compared against each other in Table 5. It is seen that $W_{1}=25\%$ is considered the best solution for both MRR and $R_{a}$, obtained by MODA followed by NSGA-II and MOALO. However, at $W_{1}=50\%$, MODA solution is marginally better than MOALO solution but comprehensively better than NSGA-II solution. At $W_{1}=75\%$, all three metaheuristics show similar performances.

### 4.2. Example 2: Optimization of Micro End Milling Parameters

#### 4.2.1. Problem Description and Formulation

A micro end milling case study from the literature [43] is considered. With the micro end milling operation, two different sizes of slots (700 μm and 800 μm) were produced in [43]. Utilizing the experimental data, four different objective functions were developed by using the same methodology adopted in example 1 above. Surface roughness ($R_{a}$) and machining time ($M_{t}$) are considered as the responses to be optimized by tuning the cutting speed (N) and feed rate (f). The developed mathematical relations for 700 μm size slot are,

(30) $$R_{a}=0.01977-0.000013N+0.00594f-0.000002Nf+0.000398f^{2}$$

(31) $$M_{t}=21.1363+0.000026N-13.9241f+0.000013Nf+2.82084f^{2}$$

Similarly, the original equations presented in [43] for 800 μm size slot are augmented as,

(32) $$R_{a}=0.0125-0.000008N+0.00678f+0.000000N^{2}+0.00003f^{2}-0.000002Nf$$

(33) $$M_{t}=20.9975+0.000133N-13.8962f+2.8211f^{2}$$

For Pareto optimization, the two objective functions can be stated as,

Objective 1 = Minimize *R_a_*

Objective 2 = Minimize *M_t_*

The optimization is performed subject to the boundary cutting conditions, which are given as,

(34) $$\begin{array}{c} 1500\;\leq \;N\;\leq \;2500\;\mathrm{rev}/\min\\ 1\;\leq \;f\;\leq \;2.5\;µm/\mathrm{rev} \end{array}$$

#### 4.2.2. Multi-Objective Optimization

**Figure 3:**
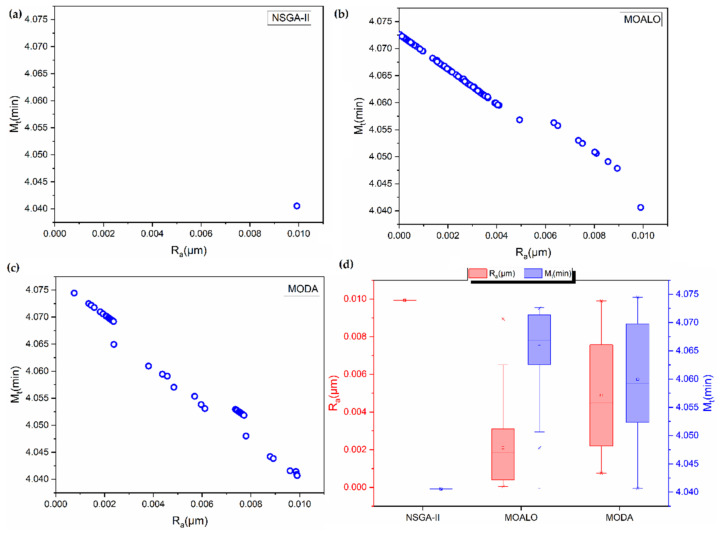
Pareto fronts for optimization of micro-milling process of 700 μm size slot using (**a**) NSGA-II (**b**) MOALO (**c**) MODA. (**d**) Box plot showing the spread of the Pareto fronts.

The Pareto fronts obtained using the NSGA-II, MOALO and MODA for micro-end milling of a 700 μm size slot is presented in Figure 3. It is seen that the NSGA-II is unable to find a Pareto front for the problem. The Pareto fronts generated by MOALO and MODA are highly discontinuous, indicating the absence of suitable non-dominated solutions. MOALO and MODA Pareto fronts have 124 and 52 non-dominated solutions, respectively. It is seen that low surface roughness is achieved by operations involving high machining time, indicating a very low cutting speed and a low feed rate. The analysis of the spreads of the Pareto fronts using box plots in Figure 3d show a better spread of MODA.

Based on the COPRAS method, the Pareto solutions of NSGA-II, MOALO and MODA are analysed to select an optimal compromise solution. It is seen from Table 6 that all the algorithms have solutions that are at par with each other. Moreover, no effect of $W_{1}$ is seen on the COPRAS selected optimal solutions, i.e., for $W_{1}=0.25,\;0.5\;\mathrm{and}\;0.75$ the same solution is predicted. This may be due to the small dimensionality of the test problem and the very limited number of non-dominated solutions in the Pareto fronts.

**Figure 4:**
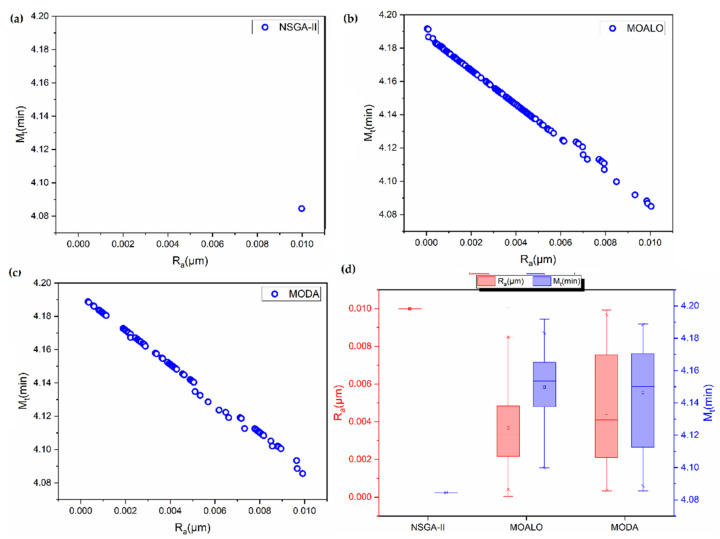
Pareto fronts for optimization of micro-milling process of 800 μm size slot using (**a**) NSGA-II (**b**) MOALO (**c**) MODA. (**d**) Box plot showing the spread of the Pareto fronts.

For the mathematical models presented in Equations (32) and (33), the Pareto solutions are shown in Figure 4. Here too, the NSGA-II is unable to find a Pareto front. The MOALO and MODA, on the other hand, generate Pareto fronts with 404 and 120 numbers of non-dominated solutions, respectively. The analysis of the solutions by COPRAS in Table 7 reveals the similar performance of NSGA-II, MOALO and MODA, as in Table 6. The least amount of average deviation is observed in the case of MOALO followed by NSGA-II and MODA. Despite the better performance of NSGA-II over MODA for this example, it should be noted that NSGA-II failed to generate a Pareto optimal front for the problem, which thereby limits options for the end user to decide from.

## 5. Conclusions

In this work, the performance of MOALO and MODA are analysed and compared with NSGA-II. NSGA-II is considered as the benchmark algorithm in this work due to its immense popularity among machining and manufacturing engineers as a tool to achieve optimal machining performance. Two micro-machining operations, namely micro-turning and micro-milling, are considered for the case studies. These methods find widespread application in modern industries for precision works. Polynomial regression is carried out and the existing mathematical relations for the test problems are made more robust by using ANOVA and a stepwise elimination method. A significant improvement in the accuracy of the mathematical models is also observed, thereby highlighting the need for ANOVA and elimination methods in such predictive modelling problems. The comparison of the metaheuristics for multi-objective optimization shows that for these types of problems in terms of computation speed MOALO > MODA > NSGA-II. Further, the Pareto front identification and generation capabilities of MOALO and MODA are found to be significantly better than NSGA-II. The COPRAS solutions for MODA was seen to be marginally better than MOALO, but both of them comprehensively outperformed NSGA-II. Thus, it may be concluded that MOALO can lead to significant cost savings in such multi-objective machining conditions by quickly and effectively identifying and generating Pareto optimal solution sets. As a future scope of this paper, more recent algorithms such as the whale optimization algorithm (WOA), multiverse optimization (MVO), spotted hyena optimizer (SHO), etc. can be applied. Hybridization by coupling multiple metaheuristics to strike a proper balance between the exploration and exploitation traits of algorithms will also be beneficial. Since often each metaheuristic is re-run multiple times to account for its inherent stochasticity, some mechanisms of leveraging the information from these multiple independent runs will be a boon.

## Figures and Tables

**Table 1:** ANOVA for the full quadratic mathematical models.

Source	MRR				$R_{a}$			
	SS	df	F-Value	*p*-Value	SS	df	F-value	*p*-Value
Model	0.0746	9	98.0800	<0.0001	0.0020	9	31.5200	<0.0001
N	0.0072	1	85.1700	<0.0001	0.0000	1	4.1000	0.0588
f	0.0246	1	291.3000	<0.0001	0.0004	1	61.6100	<0.0001
D	0.0252	1	297.5700	<0.0001	0.0012	1	176.2200	<0.0001
Nf	0.0027	1	31.7900	<0.0001	0.0000	1	0.0437	0.8368
ND	0.0033	1	38.7100	<0.0001	0.0000	1	2.0700	0.1681
fD	0.0114	1	134.5800	<0.0001	0.0002	1	22.9500	0.0002
$N^{2}$	0.0000	1	0.0114	0.9163	0.0000	1	5.0900	0.0375
$f^{2}$	0.0000	1	0.0089	0.9261	0.0000	1	0.4815	0.4971
$D^{2}$	0.0000	1	0.0453	0.834	0.0000	1	4.4300	0.0505
Residual	0.0014	17	-	-	0.0001	17	-	-
Cor Total	0.0760	26	-	-	0.0021	26	-	-

**Table 2:** ANOVA for the robust mathematical models.

Source	MRR				$R_{a}$			
	SS	df	F-Value	*p*-Value	SS	df	F-Value	*p*-Value
Model	0.0746	6	172.4065	<0.0001	0.0020	6	47.8004	<0.0001
N	0.0072	1	99.8168	<0.0001	0.0000	1	3.8989	0.062286
f	0.0247	1	342.5487	<0.0001	0.0004	1	62.4207	<0.0001
D	0.0252	1	349.6148	<0.0001	0.0012	1	179.8334	<0.0001
Nf	0.0027	1	37.2514	<0.0001	-	-	-	-
ND	0.0033	1	45.3691	<0.0001	-	-	-	-
fD	0.0114	1	157.7203	<0.0001	0.0002	1	23.4227	<0.0001
$N^{2}$	-	-	-	-	0.0000	1	5.1986	0.033711
$f^{2}$	-	-	-	-	-	-	-	-
$D^{2}$	-	-	-	-	0.0000	1	4.5198	0.04615
Residual	0.0014	20	-	-	0.0001	20	-	-
Cor Total	0.0760	26	-	-	0.0021	26	-	-

**Table 3:** Accuracy of the original and modified mathematical models.

Metric	MRR		$R_{a}$	
	Original [42]	Modified	Original [42]	Modified
R^2^	0.9811	0.981	0.9435	0.9348
Adjusted R^2^	0.9711	0.9753	0.9135	0.9153
Predicted R^2^	0.9217	0.9365	0.8532	0.8788

**Table 4:** Comparison of MOALO and MODA with NSGA-II Pareto solutions.

Metric	MOALO		MODA	
	Original	Normalized	Original	Normalized
GD	0.000012	0.000211	0.000016	0.000293
IGD	0.000056	0.000884	0.000049	0.000793
CM	0.000249	0.004399	0.000326	0.005884
SP	1.525508	1.513864	1.166575	1.126299

**Table 5:** Deviation in solutions of different metaheuristics as compared to the best solution by considering $W_{1}=0.25,\;0.5\;\mathrm{and}\;0.75$ for COPRAS calculations.

W_1_	Metaheuristic Method	COPRAS Solution		% Deviation with Respect to Best Solution		Average Deviation
		MRR	*R_a_*	MRR	*R_a_*	
0.25	NSGA-II	0.00112	0.01456	31%	0%	16%
	MOALO	0.00056	0.01453	65%	0%	33%
	MODA	0.00162	0.01459	0%	0%	0%
0.5	NSGA-II	0.06910	0.05757	0%	287%	143%
	MOALO	0.00569	0.01488	92%	0%	46%
	MODA	0.00755	0.01504	89%	1%	45%
0.75	NSGA-II	0.06910	0.05757	1%	0%	0%
	MOALO	0.06965	0.05799	0%	1%	0%
	MODA	0.06970	0.05801	0%	1%	0%

**Table 6:** Deviation in solutions of different metaheuristics as compared to the COPRAS best solution (Example 2, 700 µm slot).

Metaheuristic Method	COPRAS Solution		% Deviation with Respect to Best Solution		Average Deviation
Method	MRR	$R_{a}$	MRR	$R_{a}$	
NSGA-II	0.00992	4.04054	0.00%	0.00%	0.00%
MOALO	0.00990	4.04063	0.21%	0.00%	0.11%
MODA	0.00989	4.04070	0.30%	0.00%	0.15%

**Table 7:** Deviation in solutions of different metaheuristics as compared to the COPRAS best solution (Example 2, 800 µm slot).

Metaheuristic Method	COPRAS Solution		% Deviation with Respect to Best Solution		Average Deviation
	MRR	$R_{a}$	MRR	$R_{a}$	
NSGA-II	0.00998	4.08457	0.62%	0.00%	0.31%
MOALO	0.01004	4.08499	0.00%	0.01%	0.01%
MODA	0.00991	4.08558	1.28%	0.02%	0.65%

## Data Availability

The data presented in this study are available in the article.

## Abbreviations

ANOVA	analysis of variance
ALO	ant lion optimization
CM	convergence metric
COPRAS	complex proportional assessment
DA	dragonfly algorithm
EDM	electro-discharge machining
GA-PSO	genetic algorithm-particle swarm optimization
GD	generational distance
IGD	inverted generational distance
MCDM	multi-criteria decision making
MOALO	multi-objective ant lion optimization
MODA	multi-objective dragonfly optimization
MOPSO	multi-objective particle swarm optimization
MRR	material removal rate
MVO	multiverse optimization
NSGA-II	non-dominated sorting genetic algorithm ii
RSM	response surface methodology
SHO	spotted hyena optimizer
SP	spread
SR	surface roughness
WOA	whale optimization algorithm
